# Identification of nontuberculous mycobacteria isolated from household showerheads of patients with nontuberculous mycobacteria

**DOI:** 10.1038/s41598-022-12703-6

**Published:** 2022-05-23

**Authors:** Ji Yeon Choi, Bo Ra Sim, Youngmok Park, Seung Hyun Yong, Sung Jae Shin, Young Ae Kang

**Affiliations:** 1 Division of Pulmonary and Critical Care Medicine, Department of Internal Medicine, Severance Hospital, Yonsei University College of Medicine, 50-1 Yonsei-ro, Seodaemun-gu, Seoul, 03722 Republic of Korea; 2 Institute of Immunology and Immunological Disease, Yonsei University College of Medicine, Seoul, Republic of Korea; 3 Department of Microbiology, Institute for Immunology and Immunological Diseases, Brain Korea 21 Project for Graduate School of Medical Science, Yonsei University College of Medicine, Seoul, South Korea

**Keywords:** Microbiology, Infectious diseases

## Abstract

This study aimed to examine whether nontuberculous mycobacteria (NTM) inside household showerheads are identical to those in patients with NTM-pulmonary disease (PD) since household water is one of the potential NTM sources. Samples were obtained from 32 household showerheads of patients with NTM-PD recruited through the Pulmonary Outpatient Department at the Severance Hospital between October 2018 and October 2019. All isolates from patients with NTM-PD were diagnosed using a reverse-hybridization line probe assay based on the *ropB* gene. To determine the mycobacterial compositions, the washing fluids were collected and investigated using multiplex polymerase chain reaction assay and NTM culture; suspected microbial isolates in these fluids and culture were identified using sequencing analysis of 16S rRNA gene. NTM species causing the PD in the patients were *Mycobacterium avium*, *M. intracellulare*, *M. abscessus*, *M. massiliense*, and *M. fortuitum complex*. The mycobacteria isolated from the showerhead were *M. lentiflavum, M. gordonae, M. triplex, M. phocaicum, M. mucogenicum, M. florentinum, M. gilvum, M. llatzerense, and M. peregrinum.* However, the species identified in the showerheads did not match those of the patients. Despite NTM species in the showerheads, clinical implications in the main pathogenesis associated with the disease in the patients studied were not elucidated.

## Introduction

Nontuberculous mycobacteria (NTM) species are opportunistic pathogens responsible for progressive pulmonary disease as well as skin and soft tissue infection, lymphadenitis, and other health issues^1^. An increase in the incidence and prevalence of pulmonary disease caused by NTM has been reported worldwide^2^. In Korea, the incidence and prevalence of NTM infection increased rapidly from 2003 to 2016, particularly among women and older age groups. For reported cases of NTM infection, the incidence and prevalence rates were 17.9 and 33.3 per 100,000 populations in 2016, respectively. Additionally, the mortality rate in the NTM infected-population was higher than that in the general population^3^.

*Mycobacterium avium* complex (MAC) members, such as *M. avium* and *M. intracellulare*; *M. abscessus* complex (MABC) members, such as *M. abscessus* and *M. massiliense* are the major causative organisms of NTM-pulmonary disease (PD) and are frequently isolated from patients with NTM-PD. Recent studies have focused on identifying risk factors for both host and environmental factors associated with NTM-PD. NTM has been isolated from different environmental sources, including water, soil, food, dust, and aerosols^4,5^. Infections with NTM are caused by ingestion or inhalation of contaminated food or aerosol, and through injured skin, which indicates that a significant entry gate of NTM into a host organism is from environmental sources, such as water^6^. Previous studies have frequently identified mycobacteria in household water systems, and it has raised considerable concern among individuals susceptible to this infection. Some studies reported that patients with chronic obstructive pulmonary disease or immunodeficiency could have developed acute disorder when exposed to water contaminated with NTM^7,8^. To date this evidence has been reported mainly for MAC^4,9–13^. Thomson et al. showed that disease-causing NTM species were isolated from household water and aerosols, such as *M. avium, M. kansasii, M. lentiflavum,* and *M. abscessus*^14^. Although implicated as a potential source of disease, the mycobacterial composition and environmental predictors of showerhead-associated mycobacteria remain unresolved. Accordingly, there is a need for further epidemiological investigations of potential sources of NTM infections, including showerheads. Our study aimed to isolate and identify the NTM species present in household showerheads and compare the NTM found in biofilm with the NTM from patients with NTM-PD.

## Methods

### Sample collection and mycobacteria isolation from household showerheads

Patients with NTM-PD were recruited through the Pulmonary Outpatient Department at the Severance Hospital between October 2018 and October 2019. All patients with NTM-PD were diagnosed according to the American Thoracic Society/Infectious Disease Society of America (ATS/IDSA) 2007 guidelines^15^. For AFB smear and cultures were examined by fluorochrome staining using auramine–rhodamine and culturing in 3% Ogawa medium and mycobacteria growth-indicator tube medium (MGIT; Becton Dickson, NJ, USA). A reverse-hybridization line probe assay based on the *rpoB* gene, conducted at Seoul Clinical Laboratories (Yongin, Korea), was used for NTM species identification.

Following analysis of NTM, showerheads were collected from the homes of the patients with NTM, and a total of 32 samples were collected and 7 of the 32 samples included the shower hose. After disassembling the showerhead, the inside was wiped with a cotton swab, and the cotton swab was put in 50 mL of phosphate-buffered saline (PBS) and vortexed for 1 min. In the case of shower hoses, the front and rear entrances were parafilmed after adding 0.05% tween 20 in PBS into the shower hose. Then, the shower hose was sealed in a sterilized plastic bag and sonicated in a water bath for 5 min. After vortexing or sonicating, microbial cells in PBS were pelleted by centrifugation at 4000 rpm for 30 min. After centrifugation, the supernatant was removed, and the pellet was resuspended in 3 mL of PBS. The sample collection process from showerheads is briefly presented in Supplemental Fig. 1. The resuspended pellets were stored at 4 °C before further use. To identify the mycobacterium from natural samples, the analysis was performed using resuspended samples and cultivated of the isolates. Additionally, all resuspended samples (1 mL) were stained by Ziehl–Neelsen staining, and smear-positivity was confirmed by light microscope at 100× magnification^16^.

### Supplementation of media and growth conditions

For cultivation, the resuspended samples were decontaminated with 0.01% cetylpyridinium chloride (CPC, Sigma) and cultivated in Mycobacteria Growth Indicator Tubes (MGIT, BD BACTEC). They were supplemented with PANTA/enrichment (BD BACTEC) to obtain the following final concentrations of antibiotics in the culture medium: 40 U/mL of polymyxin, 4 µ/mL of amphotericin B, 16 µ/mL of nalidixic acid, 4 µ/mL of trimethoprim, and 4 µ/mL of azlocillin for 4 weeks (slow growers) in the MGIT system. After 4 weeks, the samples were subcultured onto 7H11 agar supplemented with 10% oleic acid-albumin-dextrose-catalase (OADC, Difco) for 4 weeks at 37 °C. After 4 weeks, they were subcultured onto Ogawa medium for 4 weeks at 37 °C^17^. Colonies of putative acid-fast bacteria were picked after 5, 10, 21, and 28 days.

### DNA preparation, multiplex polymerase chain reaction (PCR), and identification of mycobacterial target sequences

DNA was extracted from 1 mL of the resuspended sample using the conventional cetyltrimethylammonium bromide method (CTAB) as previously described^18^. Three primers were used in the PCR (IS*1311*, DT1, and 16s rRNA) according to a previously described method (Table 1)^19,20^. For amplification, each PCR mixture contained 25 μL of 2X EF-Taq PCR Smart mix (Solgent Co., Ltd. Daejeon, South Korea), 2 μL each of the 4 primer sets (all primer solutions in 10 pmol), 2 μL of DNA template, and 17 μL of water in a final volume of 50 μL. PCR was performed at 61.5 °C for 45 s for the annealing step, and followed by 30 cycles. The rest of the PCR parameters and electrophoresis were performed based on previous publications^19^. According to the multiplex PCR interpretation criteria, amplification of 16S rRNA was interpreted as identification of *M. tuberculosis*; amplification of IS*1311* or DT1 with the 16S rRNA gene was interpreted as identification of *M. avium* or *M. intracellulare*, respectively. Mycobacterium species outside the multiplex PCR target species were indicated by observation of only 16S rRNA gene amplification^19^.

**Table 1:** Primers used in the multiplex polymerase chain reaction (PCR) assay according to target *Mycobacterium* species.

Set	Genetic target	Primer sequences (forward and reverse)	Target organism(s)	Expected product size (bp)
1	16S rRNA	5′ GAGATACTCGAGTGGCGAAC 3′	All mycobacterial species	506
		5′ CAACGCGACAAACCACCTAC 3′		
2	IS*1311*	5′ TCGATCAGTGCTTGTTCGCG 3′	*M. avium complex*	600
		5′ CGATGGTGTCGAGTTGCTCT 3′		
3	DT1	5′ AAGGTGAGCCCAGCTTTGAACTCCA 3′	*M. intracellulare*	106
		5′ GCGCTTCATTCGCGATCATCAGGTG 3′		
4	*rpoB*	5′ TCAAGGAGAAGCGCTACGA 3′	All mycobacterial species	360
		5′ GGATGTTGATCAGGGTCTGC 3′		

Primer sets 1 to 4 were used for the multiplex PCR assay.

Isolates identified as other *Mycobacterium *spp. were further determined using 16S rRNA sequencing in liquid and colony samples. Sequencing reactions were performed using the BigDye (R) Terminator v3.1 Cycle Sequencing Kit from Applied Biosystems. At the end of the reaction, the dNTPs and the reactants that were not involved in the response were removed by the method recommended by Applied Biosystems. Then, the samples were loaded onto ABI Prism 3730XL DNA Analyzer to obtain sequencing results. The analyzed sequences were determined using the Sequencher 5.4.6 program from Gene Codes. The base sequence was searched in BLAST.

### Ethics approval

This research protocol was approved by the Institutional Review Board of the Severance Hospital, South Korea (IRB No. 4-2018-0444), and the study design was approved by the appropriate ethics review boards. All methods were carried out in accordance with the approved guidelines and regulations as well as in accordance with the Declaration of Helsinki. All patients gave their written informed consent.

## Results

### Baseline characteristics

A total of 32 patients were diagnosed with NTM pulmonary disease before the study period. The baseline characteristics of the patients are summarized in Table 2. There were 25 (78%) female patients, and the median age of all patients was 59 years (range 30 to 72 years). Seventeen (53%) had *Mycobacterium avium* infection, seven (22%) had *M. intracellulare* infection, and one each (3%) had *M. abscessus*, *M. massiliense,* and *M. fortuitum* complex infections. A further 5 (16%) had mixed infections. Ten of these patients were naïve for NTM treatment and the others had a history of previous or ongoing treatment for NTM-PD. Eight (25%) patients had a history of prior treatment for pulmonary tuberculosis. There were 20 patients with NTM prevalence period of 1 year or longer. Up to 6 months prior to enrollment in this study, NTM was isolated from the sputum of 30 patients.

**Table 2:** Clinical characteristics of patients with nontuberculous mycobacteria.

Clinical characteristics	Values
Age, years	59 (30–72)
Female	25 (78)
**NTM pathogens**	
*M. avium*	17 (53)
*M. intracellulare*	7 (22)
*M. abscessus*	1 (3)
*M. massiliense*	1 (3)
Mixed	6 (19)
No. of positive culture (within 6 months)	30 (93)
No. of AFB smear positive	1 (3)
**NTM treatment status**	
Naïve	10 (31)
On therapy	8 (25)
Off therapy	14 (44)
No. of NTM prevalence period > 1 year	20 (63)
**Comorbidities**	
Previous pulmonary tuberculosis	7 (22)
Bronchiectasis	24 (75)
Chronic obstructive pulmonary disease	3 (9)
Diabetes mellitus	1 (3)
Autoimmune diseases	1 (3)
Cancer	4 (13)

Values are presented as number of subjects and percentage (%) or as median (interquartile ranges). Mixed, *M. avium* and *M. fortuitum complex*; *M. avium* and *M. abscessus*; Two of *M. avium* and *M. massiliense*; *M. avium*, *M. intracellulare, M. abscessus, M. massiliense,* and *M. fortuitum complex; M. intracellulare* and *M. kansasii*. AFB; Acid-Fast Bacillus.

### Isolation of NTM from the washing fluids of showerheads using multiplex PCR

**Figure 1:**
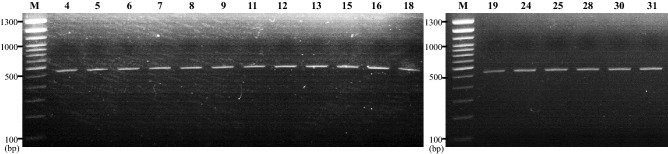
Agarose gel electrophoresis of polymerase chain reaction (PCR) amplification products using multiplex PCR primer sets on resuspended samples. Only one 500-bp band specific to the 16S rRNA gene was detected in a total of 17samples. Each line is the showerhead sample from each subject. Lane M, molecular size marker (100-bp ladder).

As described in “Methods”, samples were obtained by a swab of the surfaces of 32 showerheads, including the shower hoses from seven of the 32 showers. To isolate the NTM from the showerheads, we amplified rRNA genes from resuspended sample DNAs by PCR, using three primers (IS*1311*, DT1, and 16s rRNA, Table 1). Figure 1 shows the amplification products of isolates using multiplex PCR primer sets. Seventeen samples showed a single amplification band of 500 base pair (bp) product specific to the 16S rRNA gene for all mycobacteria. The sizes of the resulting PCR products and the groups that they identify are described in Table 1. In addition, the AFB smear was performed using resuspended samples, and identified positive results in eight samples (1+, n = 7; 4+, n = 1; negative, n = 16, and non-detected, n = 8; Table 3).

**Table 3:** Identification of clinical isolates by the multiplex polymerase chain reaction (PCR) assay and genotyping in both liquid and culture samples of each subject.

No	NTM pathogens	AFB	PCR			Resuspended samples				Culture samples			
			16s rRNA	IS1311	DT1	16s rRNA	Identities	*rpoB*	Identities	16s rRNA	Identities	*rpoB*	Identities
1	*M. intracellulare*	ND	−	−	−	ND	ND	ND	ND	ND	ND	ND	ND
2	*M. intracellulare*	ND	−	−	−	ND	ND	ND	ND	ND	ND	ND	ND
3	*M. avium*	ND	−	−	−	ND	ND	ND	ND	ND	ND	ND	ND
4	*M. avium,* *M. fortuitum complex*	ND	+	−	−	*M. triplex*	98%	Mix	Mix	ND	ND	ND	ND
5	*M. avium*	ND	+	−	−	Non-Mycobacteria	*Corynebacterium* sp. 99%	Mix	Mix	ND	ND	ND	ND
6	*M. avium, M. massiliense*	ND	+	−	−	*M. florentinum*	99%	*M. sherrisii*	97%	ND	ND	ND	ND
7	*M. avium,* *M. abscessus*	ND	+	−	−	*M. phocaicum*	100%	*M. gilvum*	97%	ND	ND	ND	ND
8	*M. avium*	ND	+	−	−	ND	ND	ND	ND	ND	ND	ND	ND
9	*M. avium*	1+	+	−	−	Low signal	Low signal	*M. gilvum*	97%	MIX	MIX	*M. gilvum*	88%
10	*M. intracellulare*	−	−	−	−	ND	ND	ND	ND	*M. gordonae*	92%	Non-Mycobacteria	*Sphingobium* sp. 93%
11	*M. avium*	−	+	−	−	*M. phocaicum*	100%	Low signal	Low signal	*M. lentiflavum*	97%	*Mycobacterium* sp.	91%
12	*M. avium*	1+	+	−	−	Low signal	Low signal	Low signal	Low signal	MIX	MIX	Non-Mycobacteria	*Rathayibacter festucae*
13	*M. avium*	4+	+	−	−	Low signal	Low signal	Low signal	Low signal	*M. gordonae*	89%	Low signal	Low signal
14	*M. avium*	−	−	−	−	*M. mucogenicum*	98%	No signal	No signal	*M. simiae* complex	92%	Non-Mycobacteria	*Gordonia terrae*
15	*M. avium*	−	+	−	−	Non-Mycobacteria	*Corynebacterium* sp.	*M. aurum*	98%	*M. lentiflavum*	91%	*M. simiae* complex	94%
16	*M. intracellulare*	−	+	−	−	MIX	MIX	*M. sherrisii*	97%	*M. lentiflavum*	97%	*M. goodi*	93%
17	*M. avium,* *M. massiliense*	−	−	−	−	*M. peregrinum*	98%	Low signal	Low signal	Non-Mycobacteria	*Gordonia* sp.	Non-Mycobacteria	*Microbacterium aurum*
18	*M. intracellulare*	−	+	−	−	*M. gilvum*	98%	*M. mucogenicum*	97%	Non-Mycobacteria	Haematomicrobium sanguinis strain	Non-Mycobacteria	*Sphingomonas paucimobilis*
19	*M. intracellulare*	1+	+	−	−	Non-Mycobacteria	*Corynebacterium* sp.	Mix	Mix	*M. gordonae*	94%	Low signal	Low signal
20	*M. massiliense*	1+	−	−	−	*M. phocaicum*	98%	Mix	Mix	*M. shigaense*	96%	Non-Mycobacteria	*Microbacterium foliorum*
21	*M. avium*	−	−	−	−	*M. gordonae*	98%	Low signal	Low signal	MIX	MIX	*Mycobacterium* sp.	91%
22	*M. intracellulare*	−	−	−	−	*M. triplex*	99%	*M. gilvum*	98%	*M. simiae* complex	91%	Non-Mycobacteria	*Micrococcus luteus*
23	*M. avium*	1+	−	−	−	*M. triplex*	97%	*M. sherrisii*	97%	*M. lentiflavum*	92%	*Mycobacterium* sp.	92%
24	*M. avium*	−	+	−	−	*M. triplex*	97%	*M. arupense*	98%	*M. lentiflavum*	99%	*M. colombiense*	94%
25	*M. avium*	−	+	−	−	*M. llatzerense*	99%	*M. phocaicum*	99%	*M. fortuitum*	97%	*Mycobacterium* sp.	96%
26	*M. avium*	−	−	−	−	*M. florentinum*	97%	*M. mucogenicum*	97%	Non-Mycobacteria	*Corynebacterium* sp.	Low signal	Low signal
27	*M. abscessus, M. fortuitum complex*, *M. avium,* *M. massiliense*, *M. intracellulare*	1+	−	−	−	Low signal	Low signal	*M. aurum*	97%	MIX	MIX	Low signal	Low signal
28	*M. avium*	−	+	−	−	*M. florentinum*	99%	*M. porcinum*	98%	MIX	MIX	Low signal	Low signal
29	*M. intracellulare,* *M. kansasii*	1+	−	−	−	*M. gordonae*	97%	Not alignment	Not alignment	ND	ND	ND	ND
30	*M. avium*	−	+	−	−	*M. mucogenicum*	98%	*M. porcinum*	97%	*M. lentiflavum*	98%	*M. godonae*	95%
31	*M. avium*	−	+	−	−	*M. mucogenicum*	98%	*M. porcinum*	97%	*M. lentiflavum*	99%	Non-Mycobacteria	*Sphingobium* sp.
32	*M. abscessus*	−	−	−	−	Mix	Mix	*M. phocaicum*	97%	*M. gordonae*	94%	*Mycobacterium* sp.	97%

ND, not done. Test not performed if the sample is contaminated or is not sufficient.

### Identification of NTM in both liquid and culture samples from the showerheads by genetic analysis

In order to identify accurate mycobacterium spp., genotype sequencing was performed in liquid and colonies of isolates sampled by PCR assay using 16s rRNA and rpoB. The species of NTM isolated from washing fluids and colonies from 32 showerheads included *M. florentinum, M. phocaicum, M. gordonae, M. mucogenicum, M. gilvum, M. llatzerense, M. peregrinum, M. sherrisii, M. aurum, M. porcinum, M. lentiflavum, M. colombiense, M. triplex, M. gordonae,* and *M. arupense*, and details are summarized in Table 3. However, none of the species from the showerhead washing fluids and cultivating colonies matched the NTM isolates from patients with NTM-PD.

## Discussion

In light of the increasing prevalence of NTM-PD without a known cause, clinicians have a substantial interest in identifying the source of the NTM infection and the number of species identified in both clinical samples and environments. In this study, NTM was isolated from the showerheads as a potential source of infection for patients with NTM-PD, and a total of 18 species (subspecies, complexes) of NTM were identified. Among them, none of the mycobacteria matched the patients’ infectious mycobacteria, but various NTM species were identified, suggesting that it could be a potential source of infection in susceptible persons.

NTM are ubiquitous in the environment and have been isolated from different environmental sources, particularly water in homes, swimming pools, whirlpool therapy baths, soils, and the workplace^21,22^. Geographic and behavioral variabilities are also associated with the incidence of NTM infection among patients^23^. Particularly, water is one of the main vehicles for NTM transmission, as confirmed by isolation of the same species from both water and patients in several studies. For example, Covert et al. reported that 35% of samples from municipal water supplies in 21 states in the United States were found to test positive for NTM^24^. In one study, strains from the water system identical to those in the patients were found in 7 (41%) of 17 patients sampled^12^. For another example, Feazel et al. showed that the showerhead environment strongly enriched for microbes that are known to form biofilms in water systems, including *Mycobacterium *spp., *Shpingomonas* spp., *Methylobacterium* spp. and others in United States^25^. Our results, and those described by various studies, show that NTM are quite frequent in household water. However, in this study, we rarely detected the major causative organisms of NTM disease, including MAC members, such as *M. avium* and *M. intracellulare*, or *M. abscessus*, *M. massiliense* from the surfaces of the showerheads. In addition, when the isolates from the patients’ showerheads were compared with those from their sputum samples, none had matching pathogens in the present study. Many factors affect the ability of NTM to adhere and form a biofilm on various materials^26^. The materials used for the showerhead, the water sources, and the type of hot-water supply system may influence the regional presence of NTM spp. on showerheads. In addition to exposure, host factors are influential in NTM infection^7,8,27–29^. According to the study by Falkinham et al.^30^, the potential impact of human activities on the ecology of NTM has also been described. Our study examined the household showerhead as a potential source of NTM. Although we did not demonstrate M*ycobacteria *spp., which was the main pathogen associated with disease in the patients diagnosed with NTM infection, we found that 17 (57%) biofilm (swab) samples collected from the showerheads of patients with NTM-PD yielded NTM, using 16S rRNA sequences. In this study, many of the isolates were identified as *M. lentiflavum* 7 (10.9%), *M. gordonae* 6 (9.4%), *M. triplex* 4 (6.3%), and *M. mucogenicum* 3 (4.7%). In our study, as in the results of several studies, *M. lentiflavum* was also the most frequently identified isolate from the house water system and could be a potential pathogen that can cause pulmonary lung disease^14,31,32^.

*M. gordonae* is known to rarely causes pulmonary lung disease due to its low pathogenicity, and when *M. gordonae* is detected in sputum, it has been considered as a contamination^33^. However, there are case reports that *M. gordonae* can cause pulmonary lung disease in immunosuppressed patients such as human immunodeficiency virus infection, steroid treatment, and organ transplant patients^34–36^. Moreover, *M. triplex* and *M. mucogenicum* be known as opportunistic infections in both immunocompromised and immunocompetent humans exposed to environmental sources and may be fatal if the infection is disseminated^24,37,38^. Accordingly, our study supports the evidence that a common indoor shower facility can be a potential source of NTM infection, which has clear relevance to public health.

In our study, the identified NTM isolates from resuspended samples and culture samples were different in several cases. There is a possibility that the specimen acquired each showerhead had a mixed microbial composition. In addition, some NTM might have hard nature to grow in cultivation. Thus, analysis of the resuspension sample and culture sample could be complementary.

The limitation of this study is that we did not consider some relevant factors, such as household water sources, estimated time since showerhead installation, usage frequency, and cleaning frequency, which may influence the ability of NTM to adhere and form a biofilm. Second, the number of acquired sample was small and most of isolated NTM were low pathogenicity species. The strength of this study is that we recovered the NTM from environmental samples by establishing methods of sample collection, processing, and cultivation.

In conclusion, our study showed the possible way of isolation and cultivation of NTM in showerhead biofilms. However, the species identified in the showerheads did not match those of the patients. Despite the identified NTM species in the showerheads, clinical implications in the main pathogenesis associated with the NTM-PD were not elucidated. Thus, further studies to explain the relationship between environmental exposure, infectious source and the route of NTM-PD are warranted.

## Supplementary Information

Supplementary Figures.

Supplementary Information.

## Data Availability

All data generated or analysed during this study are included in this published article and its supplementary information files.
