# Supplementary material for: Identification of nontuberculous mycobacteria isolated from household showerheads of patients with nontuberculous mycobacteria
Source: Sci Rep. 2022 May 23;12:8648. doi: 10.1038/s41598-022-12703-6 (PMC9127090; doi:10.1038/s41598-022-12703-6)
Supplement: Supplementary file 1 — Supplementary Figures. [file 41598_2022_12703_MOESM1_ESM.docx]

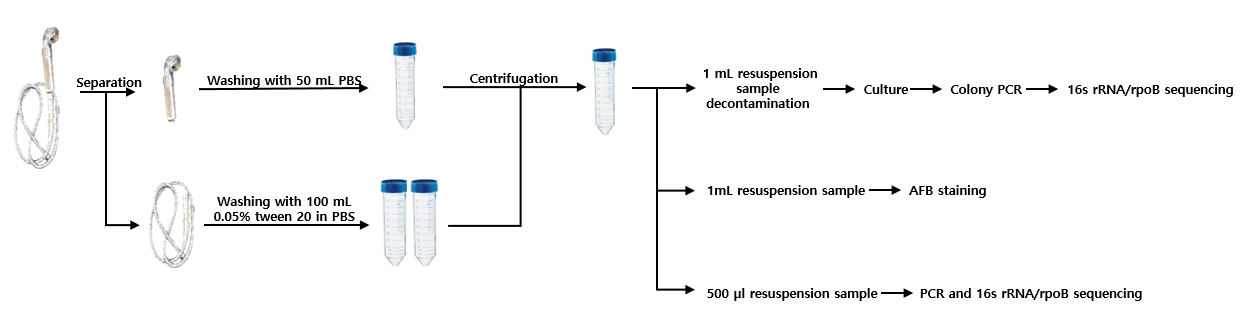


**Supplementary Figure 1. Sample collection and processing**


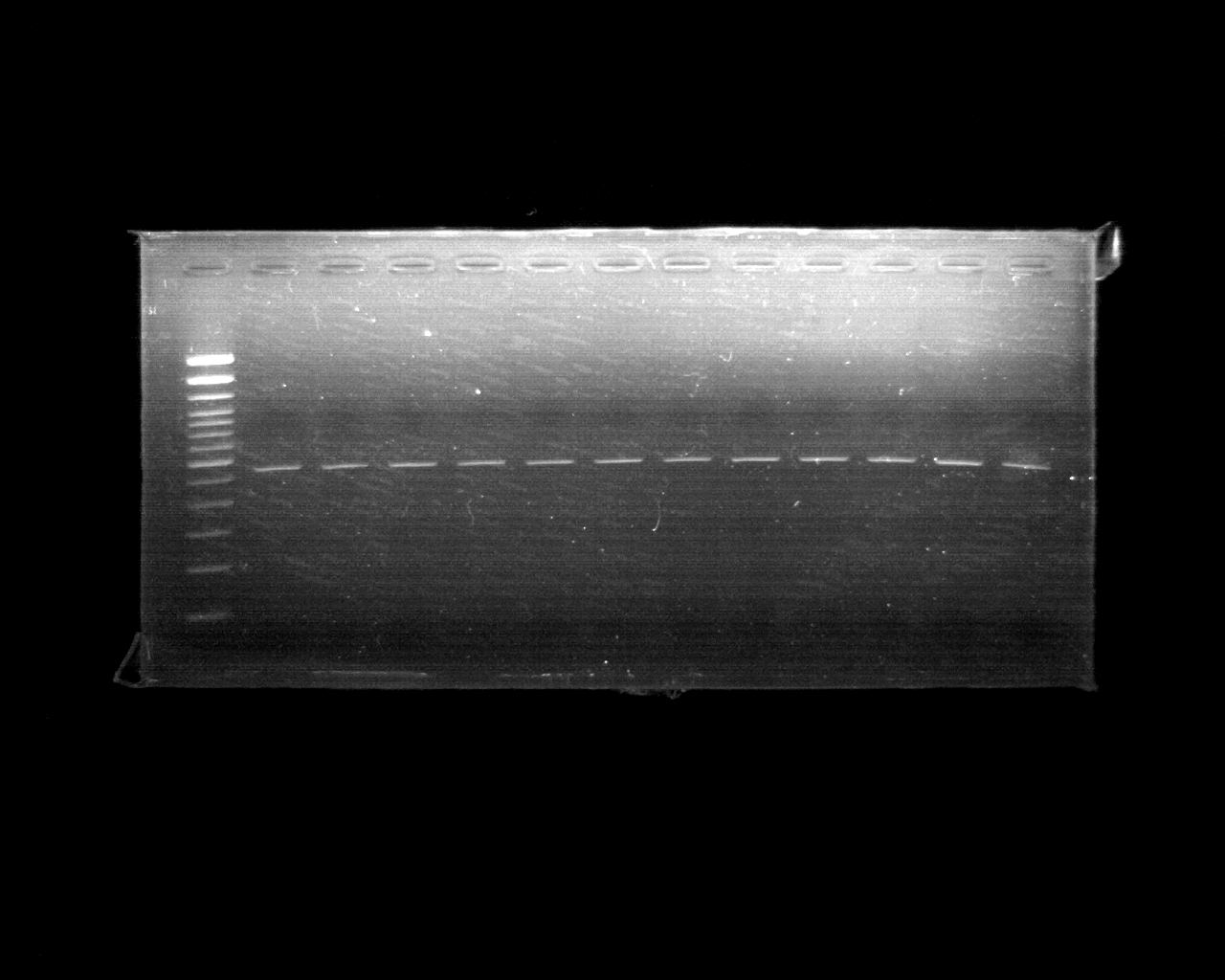

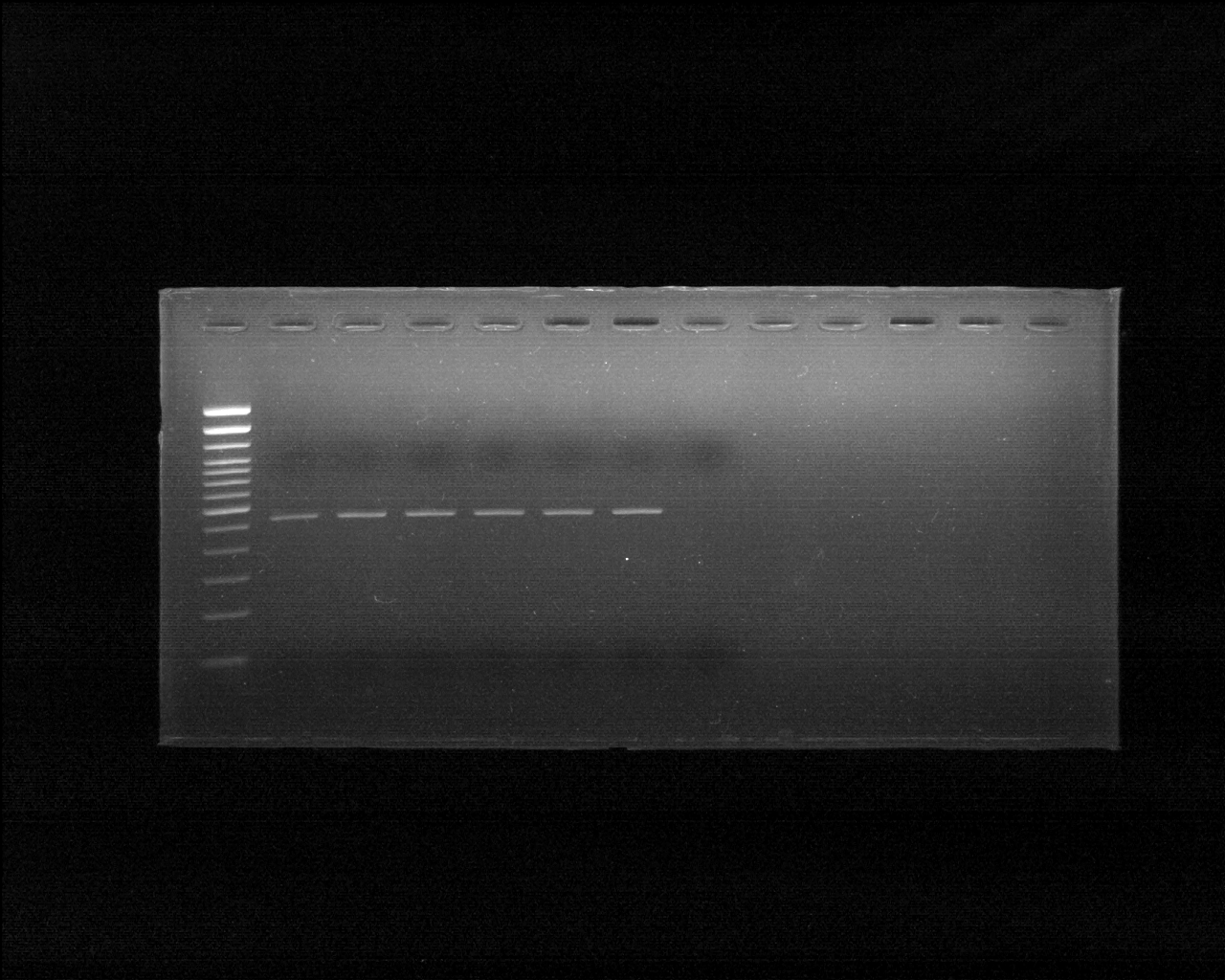


**Supplementary Figure 2. Agarose gel electrophoresis of PCR amplification products (Original images of Figure 1)**
